# The Binghampton Homœopathic Medical Association

**Published:** 1881-09

**Authors:** A. J. Clark

**Affiliations:** Secretary; Binghamton


### THE BINGHAMTON HOMŒOPATHIC MEDICAL ASSOCIATION.

THE following physicians, George F. Hand, T. L. Brown, H. S. Sloan, A. J. Clark, E. E. Synder, W. H. Proctor, of Binghamton, N. Y., and H. D. Baldwin, of Montrose, Pa., met in the City of Binghamton, at the office of Dr. George F. Hand, April 14th, 1880, and organized what has since been known as the Binghamton Homœopathic Medical Association. E. E. Snyder was chosen President, and W. H. Proctor, Secretary. There have united with this Association since its organization, Drs. J. T. Greenleaf and T. S. Armstrong, of Owego, N. Y.; H. M. Corey, Waverly, N. Y.; G. R. Bissell, Afton, N. Y.; C. F. Millspaugh, Binghamton, N. Y.; and S. S. Simmons, Susquehanna, Pa.

Dr. Armstrong is now Superintendent of the State Asylum for Chronic Insane located at this place.

We hold regular monthly meetings, selecting the subject for consideration a month in advance, and appoint some one to open the discussion. Our meetings, from the first, have been well attended, and the interest in them not only kept up but steadily increasing.

At our last meeting, July 21st, the subject for consideration was "High Potencies: Why, and how do they cure? acknowledging no drug to exist in them above the 12 cent. potency."

Dr. C. F. Millspaugh opened the discussion in a well-written paper, in which he presents a theory, somewhat peculiar, which invites criticism. In order to give the paper a wider field for criticism, it was resolved that the Secretary present it, along with some account of our Association, for publication in THE HOMŒOPATHIC PHYSICIAN of Philadelphia.

Whether we get curative results from the energy of a drug (if there can be such a thing) when the drug is all gone, certainly invites criticism. We see cures, and many remarkable cures, with attenuations above the 12 centesimal, and it must follow as a natural result that if there is no drug remaining, then there is, most certainly, some force in the shadow of moonshine. But I am inclined to believe that when the drug is all gone, the energy, the force, the curative power is all gone.

Let us hear from our *wise ones* on this speculative subject.

A. J. CLARK, M. D., Secretary.

BINGHAMTON, 7-22-'81.

